# More than a Bit of Fun: The Multiple Outcomes of a Bioblitz

**DOI:** 10.1093/biosci/biac100

**Published:** 2023-03-01

**Authors:** Sofie Meeus, Iolanda Silva-Rocha, Tim Adriaens, Peter M J Brown, Niki Chartosia, Bernat Claramunt-López, Angeliki F Martinou, Michael J O Pocock, Cristina Preda, Helen E Roy, Elena Tricarico, Quentin J Groom

**Affiliations:** Meise Botanic Garden, Meise, Belgium; Centro de Investigação em Biodiversidade e Recursos Genéticos, InBIO Laboratório Associado, in the BIOPOLIS Program in Genomics, Biodiversity and Land Planning, University of Porto, Vairão, Portugal; Research Institute for Nature and Forest, Brussels, Belgium; Zoology in the School of Life Sciences at Anglia Ruskin University, Cambridge, England, United Kingdom; Department of Biological Sciences, University of Cyprus, Nicosia, Cyprus; Autonomous University of Barcelona, Bellaterra, Catalonia, Spain; Joint Services Health Unit British Forces Cyprus, Akrotiri, Cyprus; UK Centre for Ecology and Hydrology in Wallingford, England, United Kingdom; Ovidius University, Constanța, Romania; UK Centre for Ecology and Hydrology in Wallingford, England, United Kingdom; Department of Biology at the University of Florence, Sesto Fiorentino, Italy; Meise Botanic Garden, Meise, Belgium

**Keywords:** engagement, species inventory, iNaturalist, invasive alien species, Bioblitz

## Abstract

Bioblitzes are a popular approach to engage people and collect biodiversity data. Despite this, few studies have actually evaluated the multiple outcomes of bioblitz activities. We used a systematic review, an analysis of data from more than 1000 bioblitzes, and a detailed analysis of one specific bioblitz to inform our inquiry. We evaluated five possible bioblitz outcomes, which were creating a species inventory, engaging people in biological recording, enhancing learning about nature, discovering a species new to an area, and promoting an organization. We conclude that bioblitzes are diverse but overall effective at their aims and have advantages over unstructured biodiversity recording. We demonstrate for the first time that bioblitzes increase the recording activity of the participants for several months after the event. In addition, we provide evidence that bioblitzes are effective at bringing people and organizations together to build communities of professionals and amateurs, critical for conserving and protecting biodiversity.

The word *bioblitz* (also written *BioBlitz*) first entered  the scientific vocabulary in 1996, when it was coined by Susan Rudy, of the US National Park Service, who assisted in a 24-hour event in the suburbs of Washington, DC, in the United States (Ruch et al. 2010). The event was organized by Sam Droege and Dan Roddy, and it attracted scientists and wildlife experts, employed locally either by the government or by educational establishments, such as the Smithsonian Institution (Postles and Bartlett 2018, Patuxent Wildlife Research Center 2020). The goals of this event were scientific, conservation, management, educational, public relations, and social (Patuxent Wildlife Research Center 2020), just as they are for many bioblitzes today. Indeed, it has continued to be a tool used by the National Park Service in the United States (Baker et al. 2014, National Park Service 2022).

Since the inception of the term, bioblitzes have been used all over the world as a means of gathering and sharing information about biodiversity in parks and in natural and urban areas while also often engaging large numbers of people with nature (Robinson et al. 2013, Baker et al. 2014, Postles and Bartlett 2018). Bioblitzes are diverse, but the typical event seeks to attract many people and is usually defined as a rapid assessment of the biodiversity present in a specific geographic area over a relatively short period of time (figure 1). The participants can include professional scientists and communicators, volunteer experts, and amateur naturalists, often from the local community (Lundmark 2003, Parker et al. 2018). Bioblitzes are used for rapid biodiversity assessment but also as a way of widening engagement with nature for a general audience. In the present article, in this review on bioblitzes, we use this consensus definition while recognizing that some people use the term to refer to different activities (e.g., personal bioblitzes; box 1).

**Figure 1.**
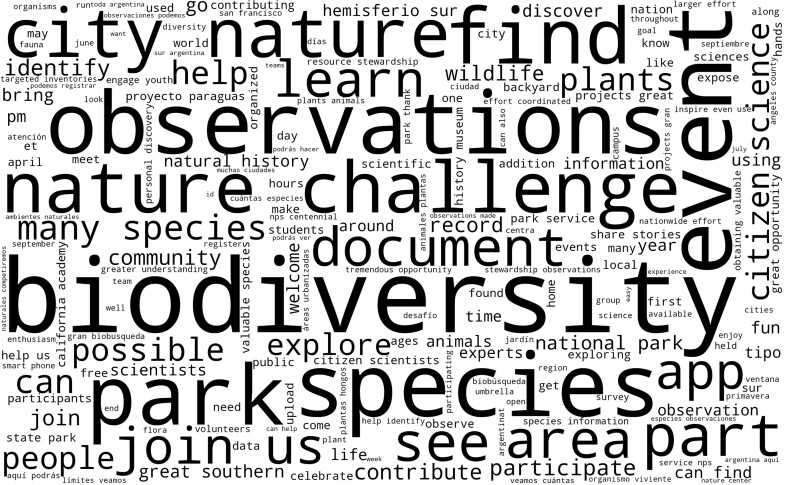
Word cloud of 1860 descriptions of iNaturalist projects with ***bioblitz*** in their title conducted between 2013 and 2020. Bioblitzes are usually pitched as fun challenges in which everyone can participate to help observe wildlife and nature by finding or documenting as many species as possible in a certain area either a city or park by, in this case, using the iNaturalist app.

Box 1. **The different kinds of events that are eponymously bioblitzes.**

The variety below shows the broad range of events but also the common themes of intensive biological recording in specific times and places. These descriptions are not exclusive; that is, a bioblitz can be both guided and place based.

**Bioblitz (general).** A rapid assessment of the biodiversity present in a specific geographic area over a relatively short period of time, in which multiple people engage and participate, either being expert scientists, naturalists or amateurs.

**Expert bioblitz.** An event, usually place based, that involves a team of professional scientists and conservation practitioners. The main aim of an expert bioblitz is to collect high-quality biological records.

**Place-based bioblitz.** A bioblitz held in a particular place at a particular time. This may or may not include public engagement.

**Dispersed (or virtual) bioblitz.** A focused, short-term event promoted via the media or social media that engages people in their location of choice. There is usually an emphasis on the total number of species recorded making it different to many other citizen science projects for nature recording.

**Personal bioblitz.** A term used by some individuals for purposeful biological recording activities undertaken by a single person.

**Targeted bioblitz.** Focus on a particular taxon. This can be a single species (e.g., an invasive alien species), insects visiting a species of flower, or a broader taxon such as birds.

**Intensive scientific survey.** A bioblitz with an emphasis on rigorous scientific data collection. The participants typically survey the site in taxon-specific teams during the whole duration of the bioblitz (cf. BioBlitz Canada 150).

**Guided bioblitzes.** An event where experts guide participants in groups; the groups may actively participate in searching for nature (so enhancing the likelihood of finding species) or may simply observe the expert undertaking recording. Guidance is provided in observing and identifying wildlife, but can also be used to ensure greater engagement, to avoid people getting lost, or to prevent them straying into conservation sensitive areas.

**Biodiversity festival.** An event offering fun and educational activities in the theme of biodiversity meant to engage people in science and nature. A biodiversity festival can be run in parallel to one or more of the bioblitz types in this box.

In bioblitzes, scientists and experts spanning different taxonomic groups often organize the event or are specifically invited to attend to contribute their survey and identification skills. Indeed, in an “expert” bioblitz, an expert team of professional scientists and conservation practitioners are the only ones invited to participate (Parker et al. 2018). However, in many bioblitzes, there is a high degree of outreach, both to experts in biodiversity recording and to inexperienced members of the public. Bioblitzes can provide an informal and fun way to create a snapshot of the variety of species that can be found in an area; they can be an opportunity for the participants to learn, share expertise, and be enthused, breaking down barriers to engagement with science (Robinson et al. 2013). Bioblitzes have become a recognized tool for environmental citizen science (DITOs Consortium 2017). They can also support outreach, where scientists communicate the importance of biodiversity in a place to the public, local communities, and policymakers (Lundmark 2003).

Bioblitzes can have many outcomes that can be divided into those for the individual participant, those for the environment, those for the community, and those for business or the economy (Robinson et al. 2013). A bioblitz always involves the collection of biodiversity data, although this is not necessarily the primary aim or outcome.

In the present article, we evaluate whether bioblitzes are suited to reach five popular outcomes indicated by bioblitz organizers: creating a biodiversity inventory for a specific time and place, discovering of new species to an area, engaging the public with natural history and research, improving the participants’ knowledge of biodiversity and the environment, and promoting an organization.

To evaluate these outcomes, we conducted a systematic review of published bioblitzes and a meta-analysis of bioblitz projects from the popular global recording app iNaturalist (www.inaturalist.org). We also describe and evaluate a case study of a bioblitz in Akrotiri, Cyprus, which the authors organized and in which they participated.

Starting as they did at the end of the twentieth century, bioblitzes have emerged in parallel to the Internet, GPS, and mobile phones. We, therefore, also show how they have evolved with information technology but still retain their original aims.

## The dimensions of a bioblitz

To understand the scope and activity of bioblitzes, we used two sources of information. First, we conducted a review of published information on bioblitzes using a search in Google Scholar for the term *bioblitz* on 31 July 2020 (for full details, see Silva-Rocha et al. 2022). We obtained information on 60 unique bioblitzes from published literature. We used the description of the bioblitz to capture data on the type (box 1), country, scale, duration, number of participants and species found, surface area, records of new species in the area, habitat, presence of a checklist, and target audience of the bioblitz. All 60 articles were then ranked on the basis of the importance—from 1 (lowest) to 5 (highest)—of the five most common aims (Baker et al. 2014, DITOs Consortium 2017, Postles and Bartlett 2018): creating a biodiversity inventory, improving knowledge of the participants, discovering new species to an area, promoting an organization, engaging the public. Thirteen papers were read and scored twice by different people. We applied Jaccard's similarity coefficient to assess agreement between the raters, because it can easily be interpreted as the percentage of agreement (Stemler 2004).

Second, we obtained summary statistics of the projects containing the word *bioblitz* in their title in iNaturalist between 2013 and 2020, with 1860 strictly fitting into the general definition of a bioblitz—that is, a short-term event from a specific place with more than one observer and at least one identifier (see Groom 2021 for code and documentation). iNaturalist is a recording platform often used in bioblitzes to inform the participants, collect wildlife records, keep score of the number of species observed, rank the participants, and so on (Unger et al. 2020). Anyone can use iNaturalist to set up their own bioblitz project, ranging from local events with a small number of participants (e.g., White Lake BioBlitz) to bioblitzes that run globally, such as the City Nature Challenge. A word cloud was created from the projects’ descriptions of the same sample of projects (Groom 2021). To assess the use of bioblitzes in the Global South, we examined the iNaturalist bioblitz data in three global regions where citizen science has tended to have lower prevalence—Africa, Asia, Latin America and the Caribbean—and looked for correlations with country-level variables such as population size and Internet penetration (for details on the methodology, see Brown et al. 2021).

To evaluate the impact of the bioblitz on the recording activity of the users—including only those people who used iNaturalist both before and after the event and users who used iNaturalist only during and after the event—a random subsample of 100 iNaturalist bioblitz projects with all their users (*N* = 3425) was selected, and recording activity, expressed as the median weekly devoted days (Ponciano and Brasiliero 2014) of each of the participants was extracted for up to a year before and a year after the bioblitz event. For each user, we calculated the difference in recording activity as the number of recording days per week before and after the bioblitz for paired weeks of the year. This approach is taken to account for seasonal variation in the detectability of species. We modeled an exponential decay function (*y* = *a ×* exp(–*bx*)) using the *saemix* R package (Comets et al. 2017), with project as a random factor. This model both fitted the data well and enabled us to calculate a half-life for the boost in activity generated by the bioblitz. For more information on the methodology, we refer you to Groom (2021) and the results in box 2.

Box 2. **An analysis of bioblitz data from the popular recording app iNaturalist.**

To explore the long-term impact of bioblitzes on participant engagement we studied the observing activity of 3378 unique participants from a random sample of 100 iNaturalist bioblitzes. We did this by comparing people's activity in the year before and the year after the bioblitz. Three-quarters (77%) of the participants used only iNaturalist during the bioblitz and a small number of people (1.5%) used iNaturalist before the bioblitz, but stopped after the event. However, 21.5% of new and existing users continued to use it after the bioblitz.

For those who continued using iNaturalist, we measured their activity as the number of days they made records per week or weekly devoted days (Ponciano and Brasiliero 2014). Then we compared their activity in the 50 weeks following the bioblitz with the same weeks in the preceding year (figure 4). Their activity was higher immediately after the bioblitz and declined toward their preexisting level of activity. According to the exponential decay model, recording activity per participant in the year after the bioblitz increased by a cumulative total of 7.4 days, and on average, the increased activity after the bioblitz halved every 12.8 weeks (decay constant = 0.054, standard error [SE] = 0.005, *n* = 38,850). Regardless of whether people were new to iNaturalist at the bioblitz or previous users of the app, a similar decay in activity was seen, although new users seemed to have a longer half-life (16.9 weeks; decay constant = 0.041, SE = 0.004, *n* = 20,950). Nevertheless, we caution overinterpretation of these results, because these are only from one app, and the proportion of new and veteran users will vary considerably between projects. The cumulative total of 7.4 days additional recording activity could have a huge impact. Even with just one observation per additional day of recording, the 113,076 people who physically engaged in iNaturalist bioblitzes in 2019 would add 180,390 additional observations after the bioblitz, equivalent to 10% of the number of observations made during all the iNaturalist bioblitzes in 2019 (*N* = 1,851,444).

By exploring these two sources of information, we found that the vast majority of bioblitzes do not have a published summary of the outcomes. However, some report the number of participants, the approaches, and the rationale behind the event. We found 60 published accounts of bioblitzes that fell within our selection criteria (Silva-Rocha et al. 2022). In our literature review, 59 of the 60 reports with the word *bioblitz* in the title (or abstract) fitted within the consensus definition, with an average duration of 31 hours. One paper describing a “personal” bioblitz of 76 days (Pollock et al. 2015) was omitted from further analyses. We also collated recommendations from these sources on how to conduct an effective bioblitz and have made these available as supplemental material (Adriaens et al. 2021).

**Figure 2.**
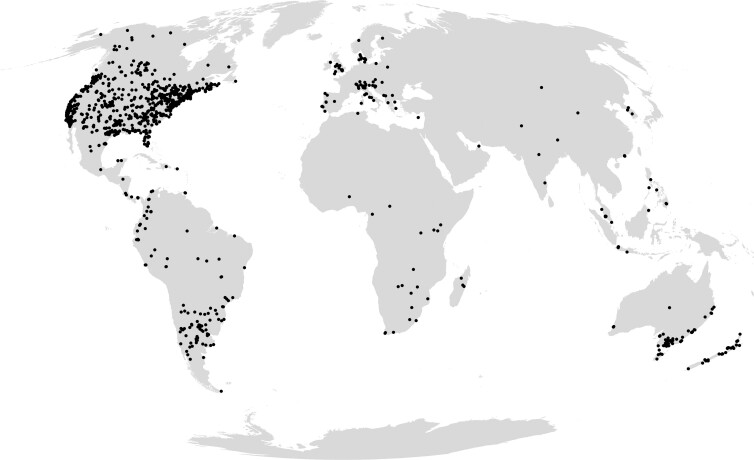
The distribution of iNaturalist bioblitz projects between 2013 and 2020 (***N*** = 1836). This map uses a Mollweide equal-area map projection.

We categorized bioblitzes from the literature into three different types of participation (*cf.* Ontaria Bioblitz, www.ontariobioblitz.ca): intensive scientific surveys, guided bioblitzes, and biodiversity festivals (box 1). More than half (57%) of the bioblitzes were guided. Most of these published bioblitzes (73%) had a local extent, with a median of 1.98 square kilometers; a fifth were regional (e.g., a state or cluster of states); and the remaining were national or global. The United States is the leading country in the number of bioblitzes (63% of published descriptions, 68% of iNaturalist bioblitzes), followed by Canada (10% and 8%, respectively; figure 2, supplemental figure S1). We did not find published accounts from Argentina, Brazil, or China, although these countries do organize bioblitzes (figure 2). Most bioblitzes were not taxon specific (63%); the remaining one-third of bioblitzes specifically targeted birds, arthropods, bats, fungi, or lichens. The number of participants varied greatly, from 10 to over 1000 (mean = 253). In terms of the output, the number of recorded species ranged from 8 to 6576 (average = 805; Silva-Rocha et al. 2022).

Most of the iNaturalist bioblitzes were run over a weekend and especially on a Saturday (supplemental figure S2). By far, the most popular months for organizing a bioblitz were April and September (supplemental figure S3). Most bioblitzes lasted less than 72 hours (76%). The average bioblitz yielded 2156 observations of 299 species, engaged 123 participants during the event, and had 154 identifiers assisting with species identifications on the iNaturalist platform (supplemental figure S4). The word cloud generated from the descriptions of these projects in figure 1 shows at a glance how bioblitzes are being promoted.

**Figure 3.**
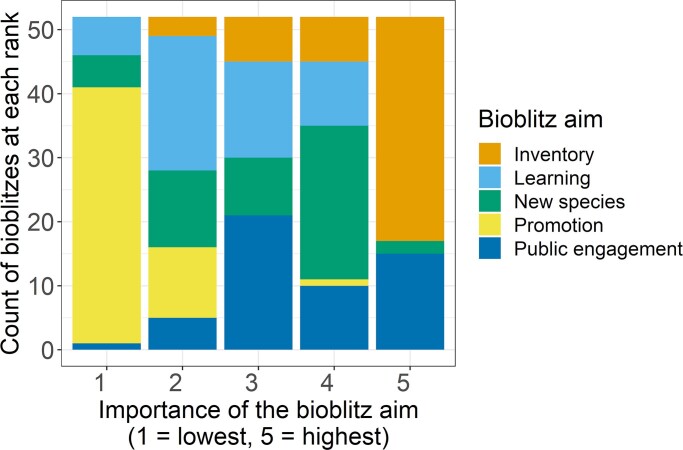
Fifty-nine published accounts on bioblitzes were screened for the five most common aims for running a bioblitz after which, for each of the publications, the aims were ranked in accordance to their importance ranging between 1 for the aim with the lowest importance and 5 for the aim with the highest importance. The following five aims were scored: inventory (i.e., creating a biodiversity inventory), learning (i.e., improving knowledge of the participants), new species (i.e., discovering new species to an area), promotion (i.e., promoting an organization), and public engagement. Either inventory or public engagement was found to be the most important aim (scores of 5) in 90% of the publications, whereas the promotion of an organization was the lowest ranked aim (scores of 1) in most of the publications.

Eight authors of this study (ISR, SM, TA, QG, NC, CP, AM, and BC) ranked each of the published bioblitzes with respect to the five bioblitz aims previously outlined. Public engagement and collecting data, either inventories or first records, were the main drivers for organizing a bioblitz in this corpus of published accounts (figure 3; Silva-Rocha et al. 2022). Although none of the published bioblitzes received the top score for learning, it is clearly an important aim; 11 publications had it ranked as the second most important outcome. Bioblitz organizers rarely mentioned the promotion of their organization to be an aim.

**Figure 4.**
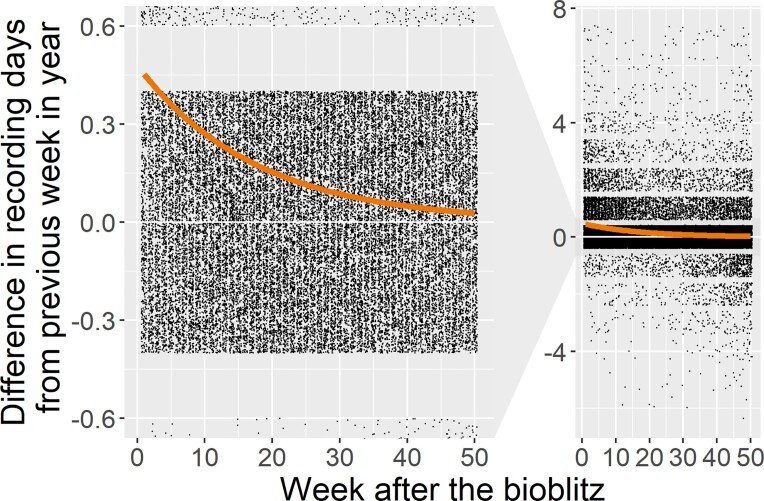
Bioblitzes trigger increased participant activity with biological recording that lasts for several weeks after the bioblitz. The difference of recording activity in days per week for 3378 recorders from 100 bioblitzes 1 year before and 1 year after the bioblitz they participated in. The ***y***-axis shows the difference in the number of recording days per week compared with the same week in the year preceding the bioblitzes (enlarged on left). Values range between −7 (i.e., if a person recorded 7 days less in the year after the bioblitz) and 7 (i.e., if a person recorded 7 days more in the year after the bioblitz) however points are jittered to make them visible. The line is fitted with a nonlinear exponential model. Pre- and postbioblitz activity were compared each week to remove any seasonal effects of recording activity and we compared recording activity expressed as the weekly devoted days rather than number of observations to help remove differences related to species abundances.

iNaturalist is used more often in medium and high income, often anglophone countries (figure 2, figure S1). For the three global regions in the Global South—Africa (30 bioblitzes in 13 countries), Asia (71 bioblitzes in 11 countries), and Latin America and the Caribbean (153 bioblitzes in 13 countries)—we found a significant correlation between the number of bioblitzes in a country and its population size (*p* < .001). There was also a significant relationship with Internet penetration (*p* < .05; Brown et al. 2021).

## Evaluation of bioblitz outcomes

The first outcome of a bioblitz is the generation of useful biological records. Most bioblitzes gather biological records that are submitted to a repository of biodiversity data (boxes 2 and 3). Bioblitzes in the United Kingdom are estimated to have contributed over 113,000 species records to local and national biodiversity recording schemes from 2006 to 2013 (Postles and Bartlett 2018). At a global scale, 2,963,742 records were contributed by 1329 bioblitz projects on the iNaturalist platform between 2013 and 2019 (box 2). The six bioblitzes ran in 2013 contributed less than 1% to the yearly total of iNaturalist records, but this increased to 377 bioblitzes, contributing 13% of the total iNaturalist records in 2019 (supplemental figure S5).

Box 3. **The Akrotiri BioBlitz.**

The Akrotiri BioBlitz took place for 24 hours in February 2019. The Akrotiri wetland is the largest natural wetland complex of Cyprus and it is famously biodiverse. It is a UNESCO Ramsar site, a BirdLife International Important Bird Area and an EU Natura 2000 Special Protection Area. The aims of the BioBlitz were to improve knowledge of the biodiversity of the Akrotiri Peninsula, to identify potential risks to the biodiversity caused by alien species, and to engage with local researchers, visiting scientists and the residents of the Peninsula. Several authors of this article participated in and organized the bioblitz, which gives us an opportunity to evaluate how well it met its aims.

**Aim 1: Improve knowledge of the biodiversity of the Akrotiri Peninsula.**

A total of 2192 observations were made on over 500 taxa. The majority of these records covered plants, insects, and birds. Most of the observations were recorded using the iNaturalist app, whereas 13% were submitted in spreadsheets and afterward published to the Global Biodiversity Information Facility (GBIF; Hadjikyriakou et al. 2019). Thirty-one species found during the bioblitz were not yet documented to occur in Akrotiri, with five of them being first records for the whole of Cyprus (Silva-Rocha et al. 2021). In this species checklist, 267 species were not yet documented to occur in Akrotiri in GBIF. These species include plants, fungi, invertebrates, birds, mammals, amphibians, and reptiles, from terrestrial, freshwater, and saline habitats. They also include some rare and underrecorded species, such as *Riccia beyrichiana, Petalophyllum ralfsii*, and *Seirophora villosa*.

**Aim 2: Early warning for invasive alien species.**

Most of the species recorded during the bioblitz were native to Cyprus (Silva-Rocha et al. 2021). Eighty-seven introduced species were recorded, of which 51 are established aliens and invasive alien species. These included well known invasive alien species such as *Acacia saligna, Gambusia holbrooki, Oxalis pes-caprae*, and *Procambarus clarkii*. The bioblitz discovered 12 new alien species for Akrotiri and added 58 new alien species to those already known from the area on GBIF. Of the 12 new taxa, some are unlikely to become invasive (e.g., *Yucca aloifolia*), others may have been overlooked native species (e.g., *Naticarius hebraeus*), and others may prove to be misidentifications and require more investigation to resolve. However, making these records available either by directly publishing them to GBIF or via the iNaturalist platform is important for further investigation and timely action against emerging problem species (Groom et al. 2015, Reyserhove et al. 2020).

**Aim 3: Engagement.**

**Figure 5.**
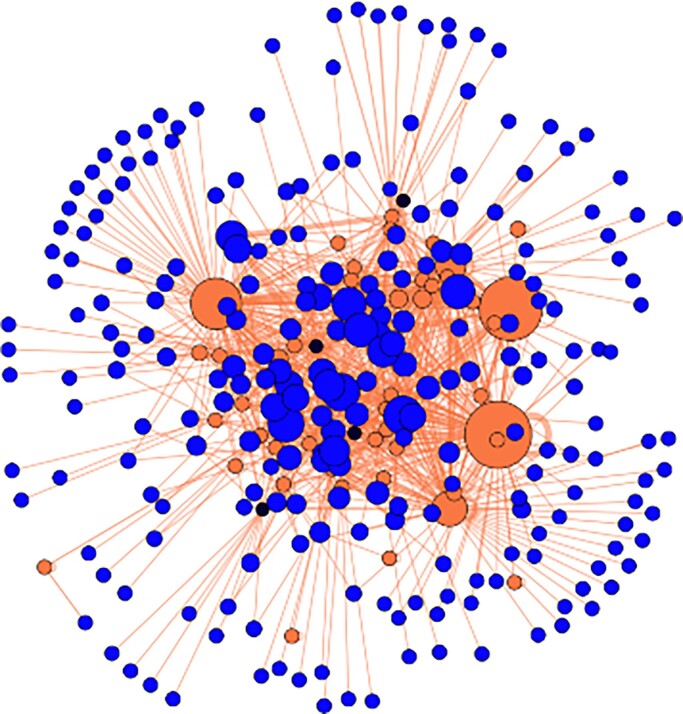
The Akrotiri bioblitz observer–identifier network was constructed using iNaturalist data on the participants engaged in the Akrotiri bioblitz (box 3) that were making observations (“observers,” the black nodes), doing identifications (“identifiers,” the blue nodes) or both (the orange nodes), and the Gephi software for the visualization (Bastian et al. 2009). The size of the nodes is proportional to the number of identifications someone did. The observer–identifier network shows that bioblitzes have the potential to engage an audience beyond the bioblitz participants by using digital platforms such as iNaturalist. Many people (blue nodes) got engaged in the Akrotiri bioblitz by identifying the records made during the bioblitz by the observers. The identifiers in the center, the bigger blue nodes in the network, engage with multiple records from the bioblitz whereas the small peripheral blue nodes are identifiers that helped in the identification of just one record.

Fifty-six observers contributed observations, although close to 100 people took part. Some of the people that did not contribute observations played supporting roles, and others contributed to observations indirectly by acting as an additional pair of eyes in a recorder team. Additional people contributed to the bioblitz by identifying the species in the records made during the bioblitz, with more than 250 people involved in the identification of these records (figure 5). Clearly, the event engaged an additional online community of people contributing through identifications, doubling the amount of people engaged during the bioblitz. Interestingly, even though most of the bioblitz observers were also involved in the identification of the records, quite a few external identifiers identified more than the people physically involved in the bioblitz (figure 5). Furthermore, although 20% of the observers were already using the iNaturalist app and continued to do so after the bioblitz, 80% of the observers were using the iNaturalist app for the first time. Of those new users, 46% continued to use the application after the bioblitz event to record their own observations.

By being a form of rapid, intense biodiversity assessment, bioblitzes can fill gaps in knowledge and provide up-to-date data that can contribute to conservation planning and management (Parker et al. 2018). These taxa may be protected species, species of conservation concern, invasive alien species, or managed species in general (Balmford and Gaston 1999, Alonso et al. 2011, Patrick et al. 2014). Parker and colleagues (2018) found that bioblitzes are cheaper, quicker, and more suited to small areas than many alternative methods of rapid biodiversity assessment. Comparing the efficacy of a bioblitz with that of a traditional expert survey in detecting herpetofauna and small mammals, Foster and colleagues (2013) found a similar efficacy of both methods to detect salamanders, snakes, and small mammals, but there was a lower detection of anurans and of rare and elusive species by the bioblitz. This was, in part, because the time limitation of the bioblitz meant that species that were not active or conspicuous were not detected, as was also reported by Ramírez Bravo and colleagues (2022). Despite limitations of bias that many types of biodiversity surveys suffer from, such as preferences for emotive taxa (Groom et al. 2021) and uneven sampling effort in time and space (Amano et al. 2016), bioblitzes are more structured than ad hoc biodiversity recording because some aspects of the survey are controlled; that is, intensity, duration, and extent are at least partially controlled. Bioblitzes might therefore generate more scientifically valuable data if basic information about the event—as a proxy of survey intensity—is provided (Kelling et al. 2019).

Expert bioblitzes, in particular, can have long-term conservation outcomes, because they generate conservation-relevant survey data, increase research capacity in undersurveyed taxa or areas, and build cross-disciplinary partnerships between people and organizations that can advance biodiversity conservation initiatives (Parker et al. 2018, Menchetti et al. 2021). This makes an expert bioblitz akin to a rapid biodiversity assessment with parataxonomists, which is an established method for biodiversity surveys in information-poor regions (Basset et al. 2000). One such expert bioblitz involving 117 taxonomic experts, over 50 students, and relevant stakeholders from the government and corporate industry has even led to the nomination of a Malaysian rainforest site as a UNESCO Man and Biosphere Reserve (Lowman et al. 2019).

Smartphone technology, including integrated GPS, high-quality cameras, and the possibility to store data and photographs locally and upload to a server, has extended the scope of mobile apps to be used in environmental and biodiversity monitoring for recording the presence and location of organisms, dating and locating different biological events (i.e., reproduction), and identifying patterns of land or seabed cover (Chandler et al. 2017, Luna et al. 2018). Examples of popular biological recording apps are iNaturalist (www.inaturalist.org), Pl@ntNet (https://identify.plantnet.org), iSpot (www.ispotnature.org), iRecord (www.brc.ac.uk/irecord) and eBird (https://ebird.org), although there are many others. Many of these apps have been used to capture the data during bioblitzes, and some, such as iNaturalist, actively facilitate it by providing a platform for data capture and online engagement tools for organizers.

Bioblitzes can discover new taxa even in well-sampled areas (Outcome 2) (e.g., Pierson et al. 2014, Nicolai et al. 2020). Even though discovering new species is not the most important motivation for organizing a bioblitz (figure 3), 63% of the papers reviewed claim discovery of taxa that are new to the area where the bioblitz was held (box 3; e.g., Cantonwine et al. 2019). Some of these newly discovered taxa are native and previously unrecorded (Maharani et al. 2022), and others are native but new to the area (colonizing due to conservation action), whereas others are newly introduced species (Silva-Rocha et al. 2022). In a 24-hour bioblitz at Christchurch Botanic Gardens and the surrounding park, for example, the participants found around 1200 different wild organisms, over a third of which were overseas introductions (Clemens and Brockerhoff 2016). During the Akrotiri (Cyprus) bioblitz, we found 12 alien species new to Akrotiri (box 3).

New records of invasive alien species can inform rapid response actions and alien species monitoring and can help in control program planning (Groom et al. 2019). Early detection and the timely eradication of invasive alien species are key to their cost-effective control (Wittenberg and Cock 2005, Vander Zanden et al. 2010, Tollington et al. 2017). Early detection requires ubiquitous and regular surveying of a broad range of species, and for this reason, citizen science has been suggested as a useful strategy for getting many eyes on the ground (Thomas et al. 2017, Parker et al. 2018, Roy et al. 2018, Dumas et al. 2020, Encarnação et al. 2021). However, one of the challenges of invasive alien species management is how to efficiently implement the detection of new arrivals. Standardized biodiversity surveys such as bioblitzes, if repeated over time, can represent a gold standard for detection of diverse taxa and patterns of introduction (Ruiz and Hewitt 2002, Ruiz et al. 2017). Existing examples are bioblitzes organized to detect yellow-legged hornets (*Vespa velutina*) on ivy (*Hedera helix*) and so help to locate the nests of this invasive species in Belgium (Schoonvaere et al. 2020) and in the coastal waters of Alaska to detect marine invaders such as the bryozoan *Schizoporella japonica* (Ruiz et al. 2017).

Bioblitzes can also play an important role in the monitoring of invasive alien species and the prioritization of invasive alien species removal actions, because these actions requires a coordinated approach that is informed by recent, accurate, and complete occurrence data, including data from nature reserves and private properties that could act as sources of reinvasion (Foxcroft et al. 2007). For example, the Texas Invasive Species BioBlitz—a 2021 bioblitz run on iNaturalist as part of the National Invasive Species Awareness Week—aimed to follow up populations of invasive alien plants by inviting the participants to revisit previously infested sites.

Most bioblitzes are not explicitly designed to detect alien species (but see Ruiz et al. 2017, Schofield 2020), but 45% of the published records specifically mention observations of non-native species (Silva-Rocha et al. 2022). A bioblitz is therefore an opportunity to raise awareness of the impacts that alien species can have on local communities (Meshaka et al. 2008, Ruiz et al. 2017), or they can be organized as part of larger public engagement and awareness initiatives (e.g., Meeus et al. 2021, Million 2016). This raised awareness in combination with the increased recording activity of the participants for many weeks after the bioblitz (box 2) can be seen as an added bonus of using bioblitzes for early warning.

Engagement of the public is included among the primary goals of many citizen science projects (Van Brussel and Huyse 2018), and this is true for bioblitzes (Outcome 3) ­(figure 3). Governmental organizations are increasingly making use of citizen science to inform aspects of the environment, including, among other objectives, engagement and raising awareness (Owen and Parker 2018, Bonney 2021), and so where bioblitzes engage public audiences, they too support these aims. However, most of the published evidence on the effectiveness of citizen science on environmental engagement is based on relatively long citizen science projects (for examples, see Rubio-Iglesias et al. 2020). For bioblitzes specifically, Postles and Bartlett (2018) showed that bioblitzes inspire positive action with their participants; however, follow-up with these participants after the event is needed to observe conversion from intent to action, such as increasing biological recording activity. To explore the impact of bioblitzes on the long-term engagement of people with biodiversity recording, we used iNaturalist data to assess the recording activity of 3378 bioblitz participants before and after the bioblitz and found a clear effect of the bioblitz: The people attending the bioblitz were likely to use iNaturalist about half a day more frequently in the week following a bioblitz than before it (taking the season into account), and this boost in activity lasted for several months (box 2).

The advent of digital recording includes the opportunity to “democratize” site-based bioblitz recording—that is, welcoming participants of different ages, genders, classes, and education levels to collect records (e.g., Aristeidou et al. 2021, Stevenson et al. 2021). The advance of technology has played a key role in facilitating the participation of volunteers in citizen science projects and have notably transformed the way bioblitzes can be organized: from the traditional structure in which scientists and volunteers survey together a given region to dispersed bioblitzes such as the City Nature Challenge, where organizers operate remotely and where volunteers may not engage physically either with organizers or even with other contributors but engage online through web-based recording platforms and social media. The dispersed nature of the City Nature Challenge has allowed it to operate during the COVID-19 pandemic.

Another benefit of uploading records to a digital platform such as iNaturalist is that identification and verification of records with photographic or audio evidence can be opened to an audience far wider than those who attended a specific event (box 3, figure 5). This enriches the observations made during bioblitzes, creates a sense of community, and extends the bioblitz experience. Through such a process of community verification, the interactions among people through associated email alerts by others invoke a memory of the past bioblitz that can motivate the participants to sustain involvement with biological recording and so provide a legacy of the bioblitz event. Engagement (e.g., the Eyal model, Eyal 2014) and gamification strategies (Dorward et al. 2017, Tang and Prestopnik 2019) have considerable potential to promote bioblitzes and retain the participation of volunteers within biological recording and more widely citizen science. This also highlights the many ways in which people can engage with a bioblitz, allowing people to be involved at many levels (e.g., Lorke et al. 2021).

Despite the many clear advantages of apps, it is also important to be mindful of their pitfalls. Not everyone has access to or has the desire to use technology. The use of apps might lead to more opportunistic recording behavior, aimed at record quantity rather than quality (Altrudi 2021). It is also likely that recording will be biased toward organisms that can easily be photographed (Adriaens et al. 2015).

From our review of bioblitzes reported in the literature, many ranked the improvement of knowledge of the participants (Outcome 4) highly, however none had learning as the highest priority objective (figures 1 and 3). Bioblitzes have been recognized as an opportunity for dialogue between experts, including scientists, and the general public, with the goal of building trust and raising awareness of environmental research (Leong and Kyle 2014, Roger and Klistorner 2016). Also, using apps and the built-in automated identification technology may support learning (Hitchcock et al. 2021), particularly in conjunction with peer and expert identification and involvement, which can include both participants and non-participants in the event (box 3; Peter et al. 2019). Rosamilia (2016) noted that learning was the most important factor in influencing satisfaction, perceptions of bioblitz success, and intentions to participate in a future event. Participants highly value the learning aspect of their attendance in a bioblitz, and both experts and non-experts feel they have learned from their participation (Roger and Klistorner 2016). This is perhaps unsurprising; it is well known that active engagement, collaboration, and respect for diverse talents are valuable tools for learning retention (Chickering and Gamson 1987). Bioblitzes meet the public's growing demand for free-choice learning—that is, the learning that individuals engage in throughout their lives when they have the opportunity to choose what, where, when, and with whom to learn, which makes a significant contribution to public understanding of science (Falk et al. 2007). Indeed, for citizen science in general, the outcomes demonstrate a high potential for learning (Peter et al. 2019, 2021), which is in line with the brain-based learning concept, which states that learning is a process that occurs through experience (Duman 2010). Following up on a campus bioblitz in Canada involving 631 students, Gass and colleagues (2021) showed that students appreciate the outdoor learning, that they believe a bioblitz provides valuable hands-on learning, that they acquired new skills in species identification, and even that they experienced an increased sense of environmental stewardship and a positive sense of place on campus.

Like any other public event, a bioblitz can increase the visibility of an organization and increase engagement with it (Outcome 5). Bioblitzes—because they are place based—can create a greater connection to a place for participants than would otherwise be the case. This can help to raise the profile of the group organizing the bioblitz, supporting its aims for promotion and building membership (Seakins and Wilkinson 2014). Although the promotion of their organization does not appear to be a primary reason to organize a bioblitz (figure 3), several papers in our literature review mentioned radio and television coverage. Bioblitzes are often branded with logos of their organizers and sponsors—for example, on promotional materials such as T-shirts and badges—and this shows that, although promotion may not be a primary driver for organizing a bioblitz, it certainly is an opportunity for that. The degree of public participation could also be used to recruit members of societies and sponsors in the context of corporate social responsibility and green agendas. In this respect, the unique place-based aspect of a bioblitz might be used to good effect.

Bioblitzes also act as brokerage events bringing together numerous institutions. This was clearly evident from our literature review (Silva-Rocha et al. 2022), where, although there was usually one coordinating institution, on average, there were an additional three coorganizing partners, but with as many as 20–30 partners represented (e.g., Telfer et al. 2015, Schofield 2020). These partners included societies, museums, botanic gardens, universities, governmental organizations, and national park administrations. Bioblitzes present an informal opportunity for people of different organizations to meet, share experiences, and, importantly, build trust (Schofield 2020).

## Opportunities for bioblitzes

Stemming from the five outcomes of bioblitzes evaluated above, we have identified three key areas of additional opportunities for bioblitz use in the future. First, because in the past, most bioblitzes have been run in high- and medium-income countries (mostly in North America and Europe) and often with quite limited participation in terms of the demographics of the participants, there is major scope for internationalization and broadening participation. Second, the very rapid technological advances and access to technologies observed in most parts of the world offer major scope for increasing the use of new technologies in bioblitzes. Third, there are opportunities for bioblitzes to have clearer links to (often local) biodiversity actions, in order to enhance their value and sustainability.

### Internationalization and designing for inclusive participation

iNaturalist is used more often in medium- and high-income, often anglophone, countries (figure 2), but bioblitzes could be adopted globally. Apps and websites, such as iNaturalist, are available in a large number of languages, which eliminate one possible barrier to participation. Mobile technology has also simplified the data management associated with biodiversity data, which allows organizers to concentrate more on the promotional and engagement aspects.

In our analysis of iNaturalist bioblitz data from countries in the Global South, it is unsurprising that there is a significant correlation between the number of bioblitzes in a country and its population size (Brown et al. 2021). But the significant correlation with Internet penetration suggests that the growth of both Internet and smartphone penetration may facilitate bioblitzes. However, Internet penetration is also correlated with gross domestic product per capita, and we should be cautious in ascribing causality (World Bank 2008). The possibility for engagement in participatory activities using mobile technology (including bioblitzes) continues to increase extremely rapidly; for example, in Sub-Saharan Africa, smartphone adoption is predicted to increase to 67% by 2025 (from 45% in 2018; Okeleke and Suardi 2021). Increased access to mobile technology and the Internet might also increase the reach of platforms such as iNaturalist and, in general, the use of environmental citizen science in, for example, species monitoring and mapping in these regions (Pocock et al. 2019). Although the priorities for citizen science will vary among regions, similar themes recur in other parts of the world, such as in Madagascar and Chile (see the case studies in Pocock et al. 2018).

In Europe, the relevance of bioblitzes has increased since the European Commission proposed that the European Union must ensure that the post-2020 global framework for biodiversity include a principle of equality (Convention on Biological Diversity 2020). This includes respect for the rights and the full and effective participation of indigenous peoples and local communities. There should be an inclusive approach, with participation of all stakeholders, including women, young people, civil society, local authorities, the private sector, academia, and scientific institutions. When considering activities such as bioblitzes that are open to many people, it may be valuable to design for the margins to increase inclusivity (Cooper et al. 2021). To implement the EU Biodiversity Strategy 2030, the links between biodiversity protection and the role of indigenous people and local communities must be strengthened (European Commission 2020). These changes should not be seen as an additional burden but a considerable opportunity to collect data in new places and to catalogue different aspects of biodiversity.

In a large study examining the participation in environmental citizen science in the United Kingdom, there were large disparities in the participation of different demographic groups. Ethnic minorities, the young, and women tended to have lower participation rates, and this was compounded in lower socioeconomic groups (Pateman et al. 2021). Callaghan and colleagues (2020) outlined that advancing biodiversity understanding in developing countries and remote areas should be a priority for citizen science. Bioblitzes have a potential role to play in this, broadening participation in environmental citizen science and ensuring that the benefits of participation are equitably spread—particularly as they provide many ways to participate. Planning for diverse participation can have enormous benefits for both organizers and participants but may require compromise or rethinking of the data-gathering objectives. Initiatives such as Black Birders Week 2021 (www.blackafinstem.com) build communities in minority groups and includes a bioblitz element. Particularly relevant to bioblitzes are the siting and timing of the events. The location of the bioblitz is crucial for including people who use public transport, and the nature of the site is important to people with reduced mobility (Pateman et al. 2021). Urban locations may facilitate inclusive participation in biodiversity citizen science through increased accessibility (Pandya 2012), both in terms of transport to sites and prior biodiversity knowledge. Similarly, the timing and duration of bioblitzes can influence participation; for example, public holidays are potentially attractive times for events but might exclude people who have religious observances.

### Technology-assisted bioblitzes

Novel technologies for biological recording have great potential for citizen science. They can be used to gather information on biodiversity that would otherwise not be available, they can increase the levels of engagement and the experience of the participants, and they may attract new audiences (August et al. 2015).

One exciting development is the use of genetic detection techniques—that is, the genetic identification of species from a range of sample types, including specimens or environmental samples (such as water or soil; i.e., environmental DNA, eDNA) and feces. This can greatly increase the completeness of rapid biodiversity assessments—for example, in protected areas and especially for difficult taxa that are otherwise unrecorded. Researchers and resource managers have been using eDNA methods to reveal and monitor endangered species, trail the emergence and spread of invasive species, and inventory biodiversity in a range of habitats, demonstrating the breadth of applications of this emerging technique (Meyer et al. 2021). Agersnap and colleagues (2022) showed that by involving citizen scientists in eDNA sampling, researchers were able to detect patterns in marine biodiversity that would have been logistically impossible to detect without the help of volunteers. Even though the participants in bioblitz events enjoy observing biodiversity itself, there is educational value and excitement in detecting biodiversity that cannot immediately be observed (Hupało et al. 2021). DNA barcoding can also significantly accelerate and facilitate the identification process when applying mass sampling techniques such as malaise traps in bioblitzes (Sobel et al. 2017). The time lag between sampling and getting the results from DNA analysis could be a challenge for engagement, although some rapid techniques could shorten this lag (Matos-Maraví et al. 2019, Meyer et al. 2021). In addition, there can be health and safety concerns associated with taking environmental samples—particularly feces.

Other approaches that have been used in citizen science and could be deployed during bioblitzes include wildlife cameras, especially for nocturnal mammals (Hsing et al. 2018), and acoustic recording devices, for bats, amphibians, birds, and insects (Gibb et al. 2019). In both cases, deploying the technology might be engaging in itself for public audiences. Then automated analyses (e.g., the BTO Acoustic Pipeline) or crowdsourced classification (e.g., MammalWeb; Hsing et al. 2018) could be used in addition to expertise during the bioblitz itself. Some technology developments could also allow for entirely different bioblitz formats. For example, Google Street View and non-Google equivalents offer possibilities for virtual bioblitzes, potentially aided by image recognition.

### Linking to action

A bioblitz is a place-based biodiversity recording activity and fills a gap between individual unstructured recording and large-scale structured recording schemes. Bioblitzes are often linked to places that have organizations owning them, providing the opportunity for a bioblitz to link more strongly to action. An in-depth study into the experiences of environmental citizen science participants shows that one of the main motivations to engage in environmental monitoring is the commitment to protect the local environment (Dunkley 2019). One way to create this strong link between records and action is to repeat recording across time so that the people can discover the impact of their records and develop a greater connection and care for the place.

## Conclusions

We have focused on one particular approach within biodiversity monitoring, that of the bioblitz. This event-based format has grown in popularity and has capitalized on social media and mobile phone technology to create something quite distinctive within biodiversity surveying. Bioblitzes have been widely used in citizen science but are not exclusive to it. They provide data at broad spatiotemporal scales and are able to collect fine-grain data suitable to address global scale conservation issues (Burgess et al. 2017).

We have shown that bioblitzes contribute a huge number of wildlife records that can be used in local and global nature conservation and serve as a trigger for further exploration of biodiversity and recording activity with the participants. We have presented the results of a literature review, of an analysis of over a thousand iNaturalist bioblitz projects, and of the bioblitz we organized ourselves in Akrotiri, Cyprus, to explore the common characteristics of bioblitzes and to make recommendations that could increase their scientific and engagement potential.

We found that bioblitzes have an added value over individual unstructured biological recording in that they allow communities to be built between experts and the public and that they are more structured and therefore yield higher-quality data. We recommend that bioblitz organizers publish their data and metadata (i.e., information on the event) in order to make the records from the bioblitz more reusable.

Smartphone applications have transformed biodiversity recording by lowering the strong dependence on experts on site during physical bioblitzes and simplifying the data management and have therefore democratized data collection. This has the added benefit of allowing socially distant bioblitzes during the COVID-19 pandemic. However, we recommend working with experts because this improves the participants’ experience and can decrease bias toward easy-to-detect and active species by making the participants aware of elusive and difficult to photograph taxa and demonstrate the (new) technologies used to detect these taxa.

There is great value in repeating a bioblitz across time for maintaining engagement of the public with wildlife recording, for connecting people to places by linking records to actions, and for obtaining more complete biodiversity inventories; therefore, we recommend that this is considered when bioblitzes are being planned. The strong link between records and action is especially obvious in invasive alien species management, a field that can clearly benefit from bioblitzes and that can use bioblitzes as a tool to increase awareness on this problem.

Bioblitzes are a great activity to promote organizations, both with the public and with other organizations, and to build trust. We recommend that organizers, whether part of a hosting organization or a community enterprise, should reach out to other organizations, collaborate, and let the bioblitz be a place to connect informally.

Although compromise is required for bioblitzes to achieve multiple outcomes, as opposed to methods with a single focus, we have shown that these outcomes work synergistically together to create events that do more than if they were focused on just one outcome. Furthermore, the parallel evolution of bioblitzes with new technologies is only likely to strengthen the ability to support these multiple outcomes. For these reasons, we see a positive future for bioblitzes and would encourage those thinking about organizing one to develop and implement their ideas. We also recommend creative exploration of the format of event-based biodiversity recording to extend its scope, because we feel that bioblitzes have a long and diverse future ahead.

## Conflict of interest

The authors declare no conflicts of interest.

## Supplementary Material

biac100_Supplemental_Files

## Author Biographical

*Sofie Meeus* (sofie.meeus@plantentuinmeise.be) and *Quentin Groom* are biologists at Meise Botanic Garden, in Meise, Belgium. *Iolanda Silva-Rocha* (irsr.14@gmail.com) is a postdoctoral researcher at the Centro de Investigação em Biodiversidade e Recursos Genéticos, InBIO Laboratório Associado, in the BIOPOLIS Program in Genomics, Biodiversity and Land Planning, at the University of Porto, in Vairão, Portugal. *Tim Adriaens* is a senior scientist at the Research Institute for Nature and Forest in Brussels, Belgium. *Peter Brown* is an ecologist in zoology in the School of Life Sciences at Anglia Ruskin University, in Cambridge, England, in the United Kingdom. *Niki Chartosia* is a marine biologist in the Department of Biological Sciences at the University of Cyprus, in Nicosia, Cyprus. *Bernat Claramunt-López* is a professor of ecology at the Autonomous University of Barcelona, in Bellaterra, Catalonia, Spain. *Angeliki (Kelly) Martinou* is an entomologist with the Joint Services Health Unit British Forces Cyprus, in Akrotiri, Cyprus. *Cristina Preda* is affiliated with Ovidius University, in Constanța, Romania. *Helen Roy* and *Michael Pocock* are ecologists at the UK Centre for Ecology and Hydrology in Wallingford, England, in the United Kingdom. *Elena Tricarico* is a lecturer in the Department of Biology at the University of Florence, in Sesto Fiorentino, Italy.
