# Correction to: More than a bit of fun: the multiple outcomes of a bioblitz

**DOI:** 10.1093/biosci/biad035

**Published:** 2023-04-11

This is a correction to: Sofie Meeus, Iolanda Silva-Rocha, Tim Adriaens, Peter M J Brown, Niki Chartosia, Bernat Claramunt-López, Angeliki F Martinou, Michael J O Pocock, Cristina Preda, Helen E Roy, Elena Tricarico, Quentin J Groom, More than a Bit of Fun: The Multiple Outcomes of a Bioblitz, *BioScience*, Volume 73, Issue 3, March 2023, Pages 168–181, https://doi.org/10.1093/biosci/biac100

In the originally published HTML version of this manuscript, figures 4 and 5 were transposed.

This error has now been corrected.

