# Supplementary figures and images for: More than a Bit of Fun: The Multiple Outcomes of a Bioblitz
Source: Bioscience. 2023 Mar 1;73(3):168–81. doi: 10.1093/biosci/biac100 (PMC10020829; doi:10.1093/biosci/biac100)

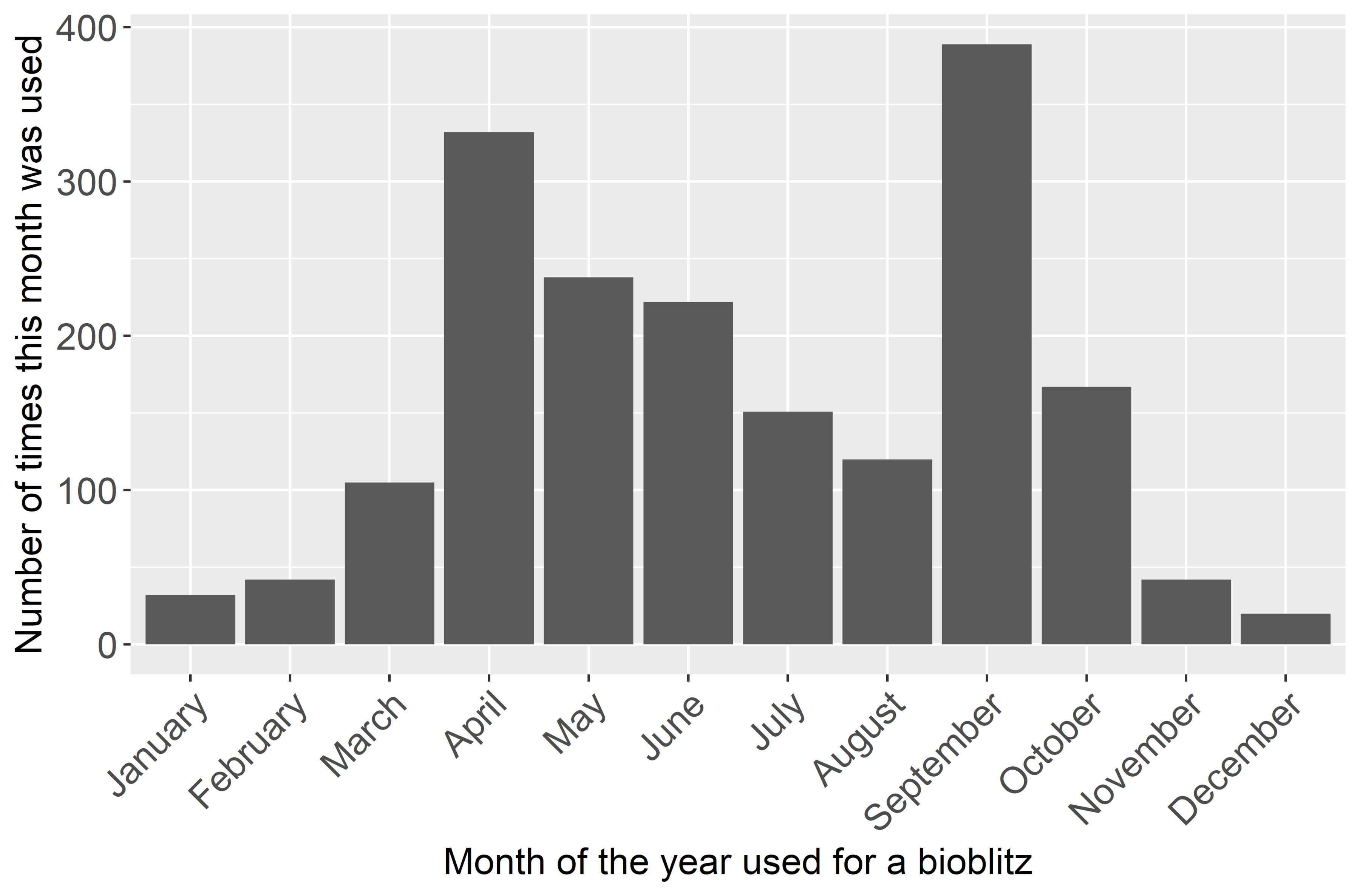

Supplement: biac100_Supplemental_Files [file biac100_supplemental_files.zip › Figure S3_600dpi.tiff]

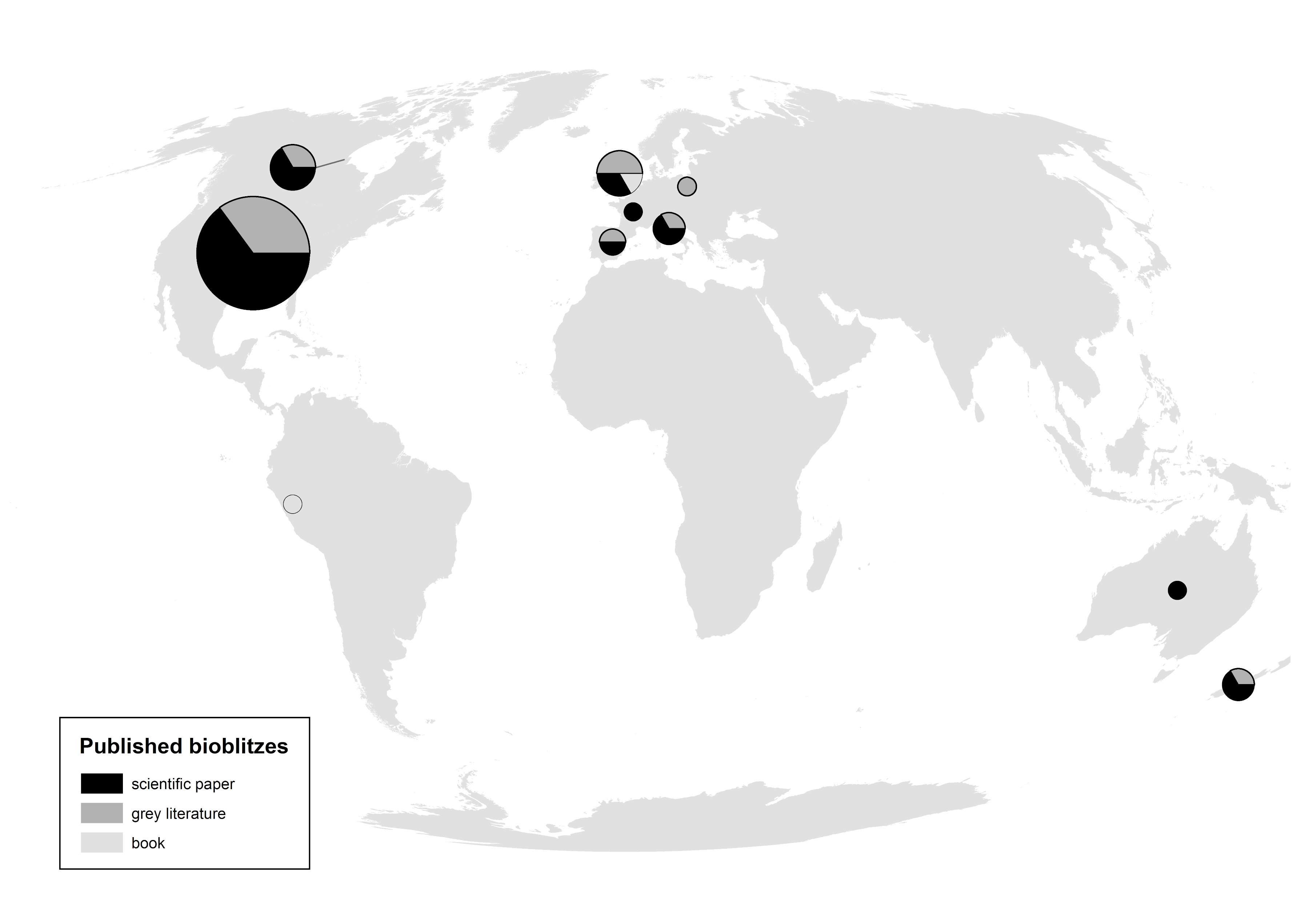

Supplement: biac100_Supplemental_Files [file biac100_supplemental_files.zip › Figure_S1_600dpi.tiff]

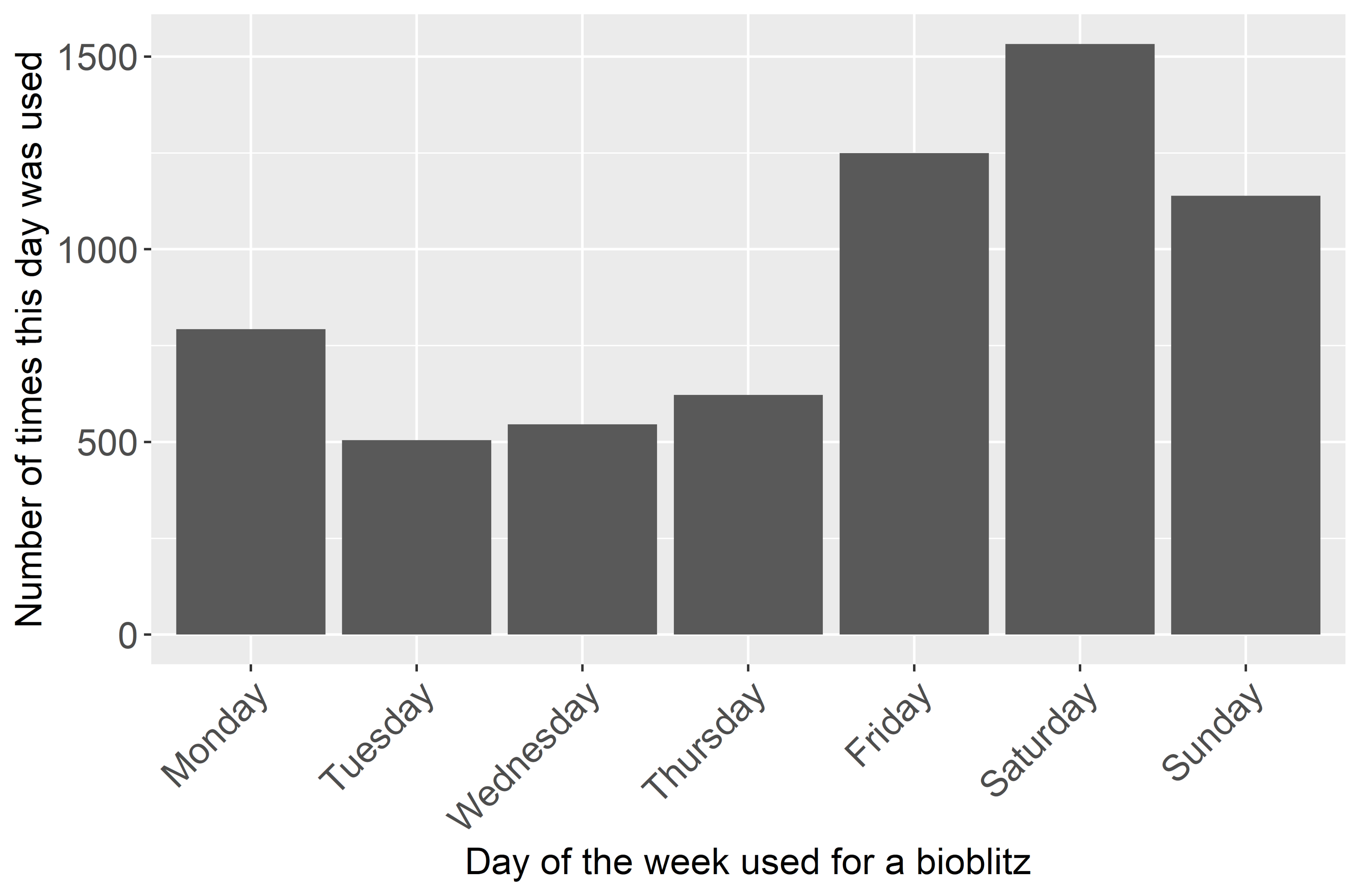

Supplement: biac100_Supplemental_Files [file biac100_supplemental_files.zip › Figure_S2_600dpi.tiff]

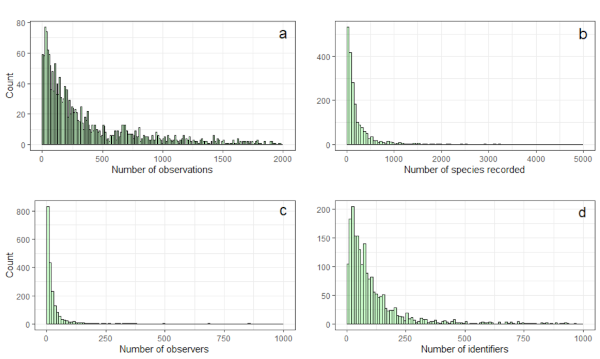

Supplement: biac100_Supplemental_Files [file biac100_supplemental_files.zip › Figure_S4_600dpi.tiff]
